# Introducing a Novel Approach for Evaluation and Monitoring of Brain Health Across Life Span Using Direct Non-invasive Brain Network Electrophysiology

**DOI:** 10.3389/fnagi.2019.00248

**Published:** 2019-09-09

**Authors:** Noa Zifman, Ofri Levy-Lamdan, Gil Suzin, Shai Efrati, David Tanne, Hilla Fogel, Iftach Dolev

**Affiliations:** ^1^QuantalX Neuroscience, Tel Aviv-Yafo, Israel; ^2^Sagol Center for Hyperbaric Medicine and Research, Assaf Harofeh Medical Center, Ramle, Israel; ^3^Sackler School of Medicine and Sagol School of Neuroscience, Tel-Aviv University, Tel Aviv-Yafo, Israel; ^4^Stroke and Cognition Institute, Rambam Healthcare Campus, Haifa, Israel

**Keywords:** brain, aging, DELPHI, plasticity, imaging, functional, network

## Abstract

**Objective:**

Evaluation and monitoring of brain health throughout aging by direct electrophysiological imaging (DELPHI) which analyzes TMS (transcranial magnetic stimulation) evoked potentials.

**Methods:**

Transcranial magnetic stimulation evoked potentials formation, coherence and history dependency, measured using electroencephalogram (EEG), was extracted from 80 healthy subjects in different age groups, 25–85 years old, and 20 subjects diagnosed with mild dementia (MD), over 70 years old. Subjects brain health was evaluated using MRI scans, neurocognitive evaluation, and computerized testing and compared to DELPHI analysis of brain network functionality.

**Results:**

A significant decrease in signal coherence is observed with age in connectivity maps, mostly in inter-hemispheric temporal, and parietal areas. MD patients display a pronounced decrease in global and inter-hemispheric frontal connectivity compared to healthy controls. Early and late signal slope ratio also display a significant, age dependent, change with pronounced early slope, phase shift, between normal healthy aging, and MD. History dependent analysis demonstrates a binary step function classification of healthy brain vs. abnormal aging subjects mostly for late slope. DELPHI measures demonstrate high reproducibility with reliability coefficients of around 0.9.

**Conclusion:**

These results indicate that features of evoked response, as charge transfer, slopes of response, and plasticity are altered during abnormal aging and that these fundamental properties of network functionality can be directly evaluated and monitored using DELPHI.

## Introduction

The ability to deal with the expanding risk of age associated brain disorders, such as vascular cognitive impairment, Alzheimer’s disease (AD), and other neurodegenerative or psychiatric disorders, the impact of which cannot be overstated, is limited by the lack of tools which enable the evaluation and monitoring of brain functional health status. These brain disorders are mostly manifested as brain network functionality changes, particularly in the early stages changing the network function. Brain functionality refers to the network topological features of connectivity, plasticity and strength which together reflect the network hierarchy and its ability to store and process information (Greenwood, 2007; Palop et al., 2007; Berlucchi and Buchtel, 2009; Feldman, 2009; Almeida et al., 2017; Kumar et al., 2017; Schulz and Hausmann, 2017). Current clinically available brain imaging provides high resolution images of the rigid brain anatomical network. Advanced technologies such as functional MRI (fMRI), or positron emission tomography can provide functional information but use indirect measurement as blood flow in high spatial but poor temporal resolution (Gore, 2003; Cherry, 2009). Therefore, these methods are insufficient for the evaluation of brain health during normal aging or age-related pathological deterioration such as mild cognitive impairment (MCI) or mild dementia (MD).

Electrophysiology is a well-established powerful tool for evaluating brain network functionality. It is used extensively in neurophysiological research, for measuring properties of brain network functionality as network effective strength of connectivity and excitation/inhibition balance that corresponds to network short term plasticity (STP) (Markram and Tsodyks, 1996; Markram et al., 1998; Bédard et al., 2004; Covey and Carter, 2015). However, clinically, it is used mostly for epilepsy and sleep monitoring, in the form of electroencephalograph – EEG, (Niedermeyer and da Silva, 2005) measuring spontaneous activity or activity related to specific task (event related potential -ERP) but is not routinely used in the clinical environment for evaluation of brain health (Shah and Mittal, 2014). Therefore, there is a great need for an ancillary tool, which enables an objective, direct, and accessible evaluation of brain network functionality in physiological terms of network connectivity, plasticity, and strength.

Transcranial magnetic stimulation (TMS) is a non-invasive brain stimulation method that allows the study of human cortical function *in vivo* (Ilmoniemi et al., 1999; Hallett, 2007; Rossini et al., 2015). TMS enables the exploration and modulation of functional neuronal networks topology with a potential therapeutic aim (Bordet et al., 2017), both in normal brain aging and in patients with degenerative or vascular dementia (Pennisi et al., 2016). Using TMS for examining human cortical functionality is enhanced by combining it with simultaneous registration of EEG. EEG provides an opportunity to directly measure the cerebral response to TMS, measuring the cortical TMS evoked potential (TEP), and is used to assess cerebral reactivity across wide areas of neocortex (Komssi et al., 2002; Nikulin et al., 2003; Rossini et al., 2015). Studies integrating TMS with EEG (TMS-EEG) have shown that TMS produces waves of activity that reverberate throughout the cortex and that are reproducible and reliable (Komssi and Kähkönen, 2006; Lioumis et al., 2009; Casarotto et al., 2010; Farzan et al., 2010; Kerwin et al., 2018), thus providing direct information about cortical excitability and connectivity with excellent temporal resolution (Kähkönen et al., 2005; Ilmoniemi and Kicić, 2010; Chung et al., 2015; Shafi et al., 2016). By evaluating the propagation of evoked response in different behavioral states and in different tasks, TMS-EEG has been used to causally probe the dynamic effective connectivity of human brain networks (Shafi et al., 2012; Kugiumtzis and Kimiskidis, 2015; Rogasch et al., 2015; Cash et al., 2017; Ferreri et al., 2017b).

Our approach, called direct electro-physiological imaging (DELPHI), is a new methodology for evaluating brain network functionality, evaluating fundamental physiological properties of brain functionality. DELPHI is a clinically available bedside tool, combining TMS-EEG and their robust scientific infrastructure, into one complete automated acquisition, and analysis system. The DELPHI software algorithm extracts direct stimulation related properties of brain network functionality in time-frequency-location. Using DELPHI, common electro-physiological features are clustered into multi-dimensional patterns of evoked network response, characterizing a profile of brain network functional pathophysiology. This neurophysiological profile includes properties of network connectivity and plasticity measured by analyzing the evoked response to changes in magnetic stimulation to specific cortical neuronal network hubs.

Aging is a normal, yet, complex biological process associated with decline in specific brain functions (sensory, motor, and cognitive) (Mora, 2013). Brain plasticity is highly important during aging, for optimal brain health (Grady et al., 2003; Kelly et al., 2006). Evidence support the understanding that network plasticity becomes less efficient with age (Rosenzweig and Barnes, 2003; Greenwood, 2007; Sambataro et al., 2010; Wang et al., 2010). Patients with early AD, reveal an abnormally suppressed efficacy of plasticity mechanisms (Greenwood, 2007; Casarotto et al., 2011; Pascual-Leone et al., 2011; Oberman and Pascual-Leone, 2013). Although common age-related diseases, such as vascular cognitive impairment and neurodegenerative disorders, are known to share patho-physiological mechanisms of alerted cortical excitability, synaptic plasticity (Bella et al., 2013; Lanza et al., 2017), and neurotransmission pathways (Bella et al., 2016), currently, there are no available tools or methods to monitor and evaluate brain health during aging.

The purpose of this work was to evaluate age dependent brain network functional changes in healthy adults using our developed DELPHI technology, providing a potential tool, and method to distinguish between normal and abnormal aging pathophysiology.

Based on the vast published data supporting the understanding that abnormal aging processes share common measurable electrophysiological features, the experimental hypothesis of this study was that using DELPHI we can characterize normal brain network functional aging, and differentiate it from abnormal aging defined in this study as MD.

## Materials and Methods

### Clinical Data Collection and Analysis

This study was carried out in accordance with the recommendation of “Assaf-Harofeh” Medical center review board. Protocol was approved by the local institutional “Ethical Committee.” All subjects gave written informed consent in accordance with the Declaration of Helsinki. All participants underwent the exact same TMS-EEG stimulation and recording protocol.

### Subjects

Four study groups were included in the study, defined as follows: (a) Healthy young, 25–45 years old (*N* = 30; mean age: 35, stdv: 6.6); (b) Healthy adults, 50–70 years old (*N* = 30 mean age: 61; stdv: 5.9); (c) Healthy elderly, over 70 years old; (*N* = 17; mean age: 75.4; stdv: 5.6); (d) MD subjects, over 70 years old (*N* = 20; mean age: 75.2, stdv: 4.3). Statistics of each study group is described in Tables 1, 2. Inclusion criteria for the healthy subject groups were as follows: (1) No neurological or psychiatric disorder documented in medical history or self-report. (2) Absence of any significant abnormal findings in MRI scan such as brain tumors, subdural hematoma, and other brain structural lesion. (3) No central nervous system (CNS) directed prescribed medication treatment. (4) A global index score and memory (verbal and non-verbal) of 95 or above (normalized to age related population) in computerized testing. MD inclusion criteria were defined as follows: (1) A clinical diagnosis of probable AD Dementia (McKhann et al., 2011). (2) Montreal cognitive assessment (MoCA) score between 11 and 22 as evaluated by neuropsychologist (Nasreddine et al., 2005; Saczynski et al., 2015). (3) Computerized testing index score of at least 1.5 standard deviations (STDV) below age related norm in verbal and non-verbal memory score and at least in one out of 3 additional computerized tests (Attention/Information Processing/Executive Function). (4) Absence of other unrelated neurological or psychiatric disorder documented in medical history or self-report. (5) Absence of any unrelated significant abnormal findings in MRI scan such as brain tumors, subdural hematoma, and other brain structural lesion. Tables 3, 4 summarize study groups computerized cognitive score per cognitive domain. Mean MoCA score of the MD subject group was 16 ± 4.

**TABLE 1:** Statistical distribution of study groups.

	**Total N of subjects**	**N of male**	**N of female**	**N right- handed**	**N left- handed**	**Mean age (year)**	**Std**
Mild dementia (>70)	20	11	9	19	1	75.2	4.3
Healthy (>70)	17	12	5	16	1	75.4	5.6
Healthy (50–70)	30	23	7	29	1	61	5.9
Healthy (25–45)	30	16	14	28	2	35	6.6

*Each row represents different study group.*

**TABLE 2:** Statistical differences between ages of study groups.

	***p* value**
Mild Dementia (>70)\Healthy (>70)	ns/*p* = 0.77
Healthy (>70)\Healthy (50–70)	*p* < 0.0001
Healthy (50–70)\Healthy (25–45)	*p* < 0.0001

*Each row represents two study groups, right column displays *t*-test *p* values.*

**TABLE 3:** Statistical distribution of study groups computerized testing scores.

	**Global score**		**Memory**		**Executive**		**Attention**		**Information**		**Motor skills**	
					**function**				**processing speed**			
	**Mean**	**STD**	**Mean**	**STD**	**Mean**	**STD**	**Mean**	**STD**	**Mean**	**STD**	**Mean**	**STD**
Mild dementia (>70)	81	11	71	8	85	13	80	19	96	7	91	17
Healthy (>70)	108	7	109	5	113	9	103	8	108	14	110	7
Healthy (50–70)	105	6	102	9	108	9	104	8	107	11	107	5
Healthy (25–45)	103	10	103	6	104	10	101	9	101	17	107	12

*Each row represents a different group in the study, each column represents a different cognitive domain that was evaluated.*

**TABLE 4:** Statistical differences between study groups computerized testing scores.

***P* values**	**Global score**	**Memory**	**Executive function**	**Attention**	**Information processing speed**	**Motor skills**
Mild dementia (>70)\Healthy (>70)	0.000	0.000	0.000	0.005	0.130	0.005
Healthy (>70)\Healthy (50–70)	0.096	0.863	0.247	0.531	0.864	0.258
Healthy (50–70) \Healthy (25–45)	0.367	0.529	0.221	0.237	0.314	0.877

*Each row represents two study groups that were compared, each column represents a different cognitive domain that was evaluated and displays *t*-test *p* values.*

All subjects underwent a brain MRI scan 1–2 weeks before DELPHI evaluation. Imaging was performed with a 3 Tesla system (20 channels, MAGNETOM Skyra, Siemens Medical Solutions). The MRI protocol included T2 weighted, T1 weighted, FLAIR, and susceptibility weighted imaging (SWI) sequences. All scans were evaluated at “Assaf-Harofeh” medical center by a neuro-radiologist. Assessment of cognitive functions was performed by trained neuropsychologists using the MoCA test (Nasreddine et al., 2005) and NeuroTrax *Mindstreams* Mild Impairment Battery computerized BrainCare cognitive battery tests (NeuroTrax Corp., TX, United States) (Dwolatzky et al., 2003).

### TMS-EEG

Transcranial magnetic stimulation was performed with a MagPro R30 stimulator (MagVenture, Denmark) and an MCF-B65-HO figure-8 Coil (MagVenture, Denmark). 32-channel EEG data were obtained using two 32-channel TMS compatible BrainAmp DC amplifiers (5 kHz sampling rate; ±16.384 mv measurement range; analog low pass filter 1 kHz; Brain Products GmbH, Germany). These were attached to the Easy EEG cap (EasyCap GmbH, Germany) with Ag-AgCl electrodes. Electrode impedances were kept below 5 kOhm. The reference and ground electrodes were affixed to the ear lobes. EEG data were recorded using a BrainVision Recorder software (Brain Products GmbH, Germany). All data were pre-processed and analyzed using our developed fully automated DELPHI algorithm and implemented in MATLAB (R2016b, The Mathworks Inc., MA, United States).

### Experimental Procedure

Transcranial magnetic stimulation coil was positioned over the left cortical motor (M1) region, at 45° toward the contralateral forehead according to guidelines (Rossini et al., 2015). Each TMS-EEG run entailed 420 pulses (biphasic pulses at 280 ms pulse width) at ranging intensities, from 25 to 60% of the maximal device intensity of stimulation varied in frequencies from 0.1 Hz up to 20 Hz. A thin (0.5 mm) foam pad was attached to the TMS coil to minimize electrode movement and bone-conducted auditory artifact. Participants were instructed to keep their eyes closed throughout the examination to reduce ocular artifacts. The operator of the system conversed with subjected between the short stimulation protocol blocks in order to avoid drowsiness. Electrode were grouped for statistical purposes: Frontal, F3, F5 -ipsilateral and F4, F6- contralateral to stimulation. Parietal, C3, C5, CP1 -ipsilateral and C4, C6, CP2- contralateral to stimulation. Temporal CP5, CP3, CF5 -ipsilateral and CP6, CP4, FC6- contralateral to stimulation. Occipital cortex, O1, PO3 -ipsilateral and O2, PO4- contralateral to stimulation.

### Sham Stimulation

For sham TMS stimulation a realistic sham was performed by spacing the TMS coil in order to maintain auditory, pressure and tactile parameters with reduced magnetic field (Veniero et al., 2009; Zanon et al., 2013; Gordon et al., 2018). The figure of 8 coil was placed over the left cortical motor (M1) region in the exact same orientation as for non-sham stimulation. After placement, the coil was moved 3 cm away from the scalp and a silicone cube (10 cm × 3 cm) filled with artificial cerebral spinal fluid (aCSF) (Dolev et al., 2013) was placed between scalp and TMS coil. Stimulation protocol (duration, intensities, and frequencies) was maintained the same as in non-sham (Supplementary Figure 1).

### DELPHI Analysis

Direct electrophysiological imaging analyzes the regional and network TMS evoked EEG pattern of response to single and history dependent events. A single TMS pulse delivered over the primary motor cortex (M1) results in a sequence of positive and negative EEG peaks at specific latencies (i.e., N45, P60, N100, and P180; negative peaks 45 and 100 msec after stimulation, positive peaks 60 and 180 msec after stimulation, Supplementary Figure 2). This pattern of response indicates synaptic activity, specifically the Glutamate-excitatory and Gamma-aminobutyric acid (GABA)-inhibitory transmission balance (Du et al., 2018). It is considered that the P60 peak represents activity of α1-subunit-containing GABA-A receptors whereas the N100 represents activity of GABA-B receptors (Premoli et al., 2014a). These TMS-evoked cortical potentials last for up to 300 msec in both the vicinity of the stimulation, as well as in remote interconnected brain areas that reflect long term changes in cortical network excitation-inhibition balance, referred to as brain network plasticity (Bonato et al., 2006; Daskalakis et al., 2008; Fitzgerald et al., 2008; Casarotto et al., 2010; Premoli et al., 2014a, b). Changes in TMS evoked short term plasticity measurements provide important insights into cortical processing both in health (Massimini et al., 2005; Ferrarelli et al., 2010) and disease (Daskalakis et al., 2002; Julkunen et al., 2008; Kaster et al., 2015; Manganotti et al., 2015; Ferreri et al., 2017a). All data processing is performed automatically by the DELPHI software algorithm.

### DELPHI Architecture

**FIGURE 1:**
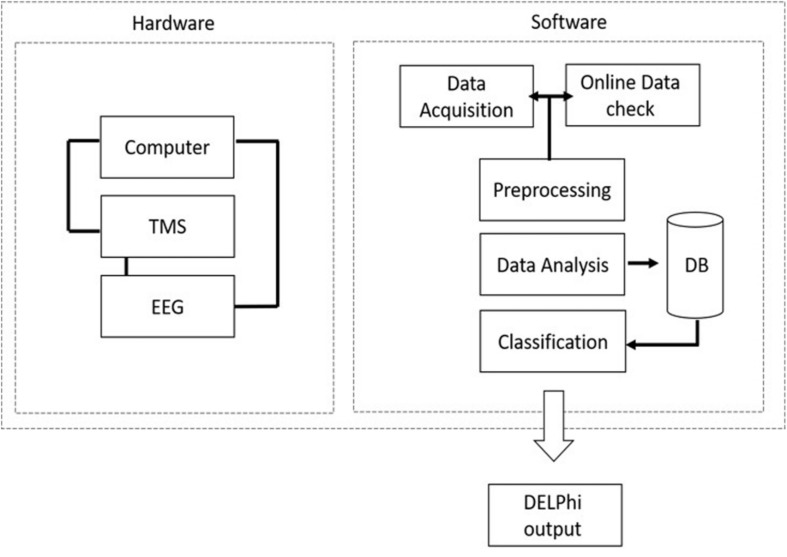
DELPHI system hardware and software architecture: DELPHI software algorithm architecture is divided into five layers.

Direct electrophysiological imaging is composed of customized integrated hardware devices (TMS and EEG), combined with an automated acquisition and analysis software (Figure 1).

Direct electrophysiological imaging software algorithm architecture is divided into five layers, as outlined in Figure 1A. Data acquisition: automated data collection. A fixed stimulation protocol of TMS in varying intensities and frequencies, introduced to specific pre-determined locations on the skull, ensuring accuracy of the acquired TEP data. (B) Online data check: automated and continuous evaluation of the collected data quality for optimal collection at minimum acquisition time. The online data check ensures a continuous online feedback of data quality. (C) Data pre- processing: automated rapid cleaning of data following acquisition. (D) Data analysis and features extraction: measured signal features are extracted and calculated for determining the relevant electrophysiological parameters of DELPHI physiological profiling. (E) Classification of population subgroups. DELPHI electrophysiological parameters constitute the subject network physiological profiling, which is displayed as numeric raw values. The reliability of DELPHI as a state and disease classification tool increases with the growth in the quantity of collected neuro-physiological biomarkers data.

### DELPHI Physiological Network Profile Analysis

**FIGURE 2:**
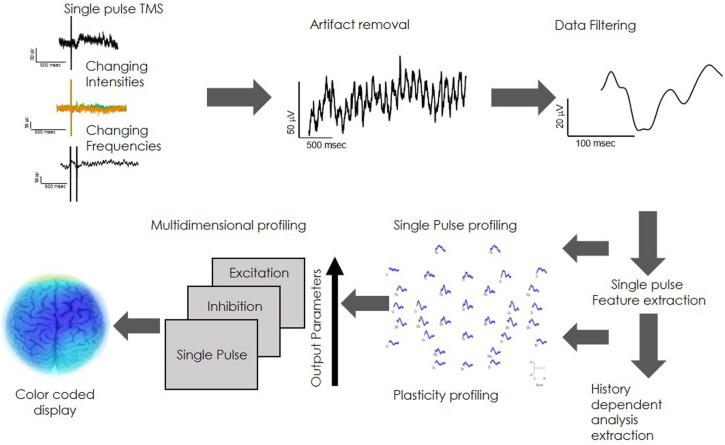
Outline of the DELPHI functional network analysis.

Direct electrophysiological imaging profile, characterizing brain network functionality, analyzes the physiological features of the local brain response to stimuli. The analysis regard two fundamental features of brain physiology: (1) Single pulse. This refers to the evoked response to a single TMS pulse in varying intensities, calculated as the local Input/output curve (Supplementary Figure 2A). Evoked response is represented as a collection of amplitudes, slopes and latencies (P60-N100 slope is referred to as the early slope and the N100-P180 slope as the late slope) (Rogasch et al., 2015; Tremblay et al., 2019). (2) Network plasticity. Refers to frequency dependent changes in evoked response, an attribute that expresses the history dependency of the network. Introducing high frequency of stimulation (>=20 Hz) evokes excitation of network response (Maeda et al., 2000; Garcia-Toro et al., 2006) while low frequency (>=5 Hz) evokes inhibition of the regional network response in a mechanism that may be similar to long term depression -LTD (Muellbacher et al., 2000; Fitzgerald et al., 2006; Supplementary Figure 2B). Data acquisition is performed automatically by introducing a sequence of stimuli in changing intensities and frequencies (Figure 2A) followed by a bilayer data cleaning step of TMS artifact removal and data filtering (Figures 2B,C). Average response features of charge transfer, slopes and latencies are extracted (Figure 2D), providing the single pulse and plasticity profile of network functionality. These physiological parameters are unified into one multidimensional neuro-physiological DELPHI profile of brain network functionality (Figure 2E). Cortical network values may be translated into pseudo-colored coded image describing brain network functionality (Figure 2F).

Reproducibility test was performed on collected and analyzed parameters. Results demonstrate high reliability and reproducibility of the DELPHI analyzed physiological parameters displaying reliability coefficient (r) of 0.87 and 0.94 (Supplementary Figure 3).

### Statistical Analysis

Statistical data analysis was performed using GraphPad Prism 7. Reproducibility measures were compared by Pearson’s correlation. Error bars shown in the figures represent standard error of the mean (SEM). The number of subjects is defined by *N*. One-way ANOVA analysis with *post hoc* Tukey was used to compare subject groups. Student’s un-paired *t*-test was used to compare two groups. ^∗^*p* < 0.05; ^∗∗^*p* < 0.01; ^∗∗∗^*p* < 0.001, ^∗∗∗∗^*p* < 0.0001, ns, non-significant.

## Results

### Evaluation of Age Dependent Changes in Network Connectivity

Brain Network Connectivity and Coherence Are Indicators of Network Health and Function. The concept of using TMS-EEG for evaluating brain neuronal network by monitoring its response has been described in numerus papers and has been shown to provide clinical evidence for functional brain network pathophysiological deterioration (Grady et al., 2003; Wang et al., 2010; Legon et al., 2016).

**FIGURE 3:**
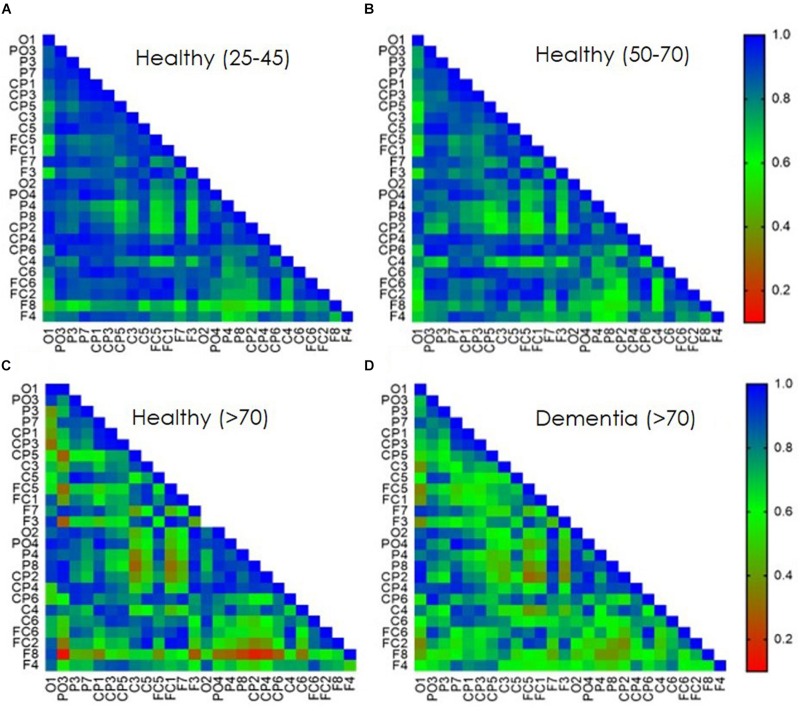
Age dependent TEP-connectivity matrixes analysis. Pearson’s correlation coefficient of bi-lateral EEG sensors single pulse evoked response. Data presents averaged population response **(A)** 25–45 years old healthy group. **(B)** 50–70 years old healthy group. **(C)** Over 70 years old healthy group. **(D)** Over 70 years old diagnosed with Mild Dementia (MD). Correlation values are presented as consecutive color-coded bar. blue, high correlation; red, low correlation.

Direct electrophysiological imaging analysis of single pulse response is displayed as connectivity matrixes (Pearson’s r) of averaged age groups (Figure 3). A decrease in signal coherence is observed with age, young 25–45 matrix (Figure 3A) display high correlation values which are slightly decreased in the healthy 50–70 group (Figure 3B) and decreases further in the over 70 healthy group (Figure 3C). A significant decrease is observed for young 25–45 and over 70 healthy group between left and right parietal and temporal areas (*p* < 0.01), but not between frontal right and left areas. A significant decrease is also displayed for frontal and parietal areas (*p* < 0.01), frontal and temporal areas (*p* < 0.01), and frontal vs. occipital areas (*p* < 0.01). There is also a significant decrease in *r* values for the 50–70 and over 70 healthy group for frontal connectivity between frontal and parietal areas (*p* < 0.01), frontal and temporal areas (*p* < 0.01), and frontal vs. occipital areas (*p* < 0.01). When comparing the MD patients, over 70 years old, with healthy over 70 controls (Figure 3D) a pronounced decrease in frontal inter-hemispheric connection is displayed (*p* < 0.01) as well as a decrease in connectivity values between frontal and contralateral parietal, temporal, and occipital areas (*p* < 0.01).

**FIGURE 4:**
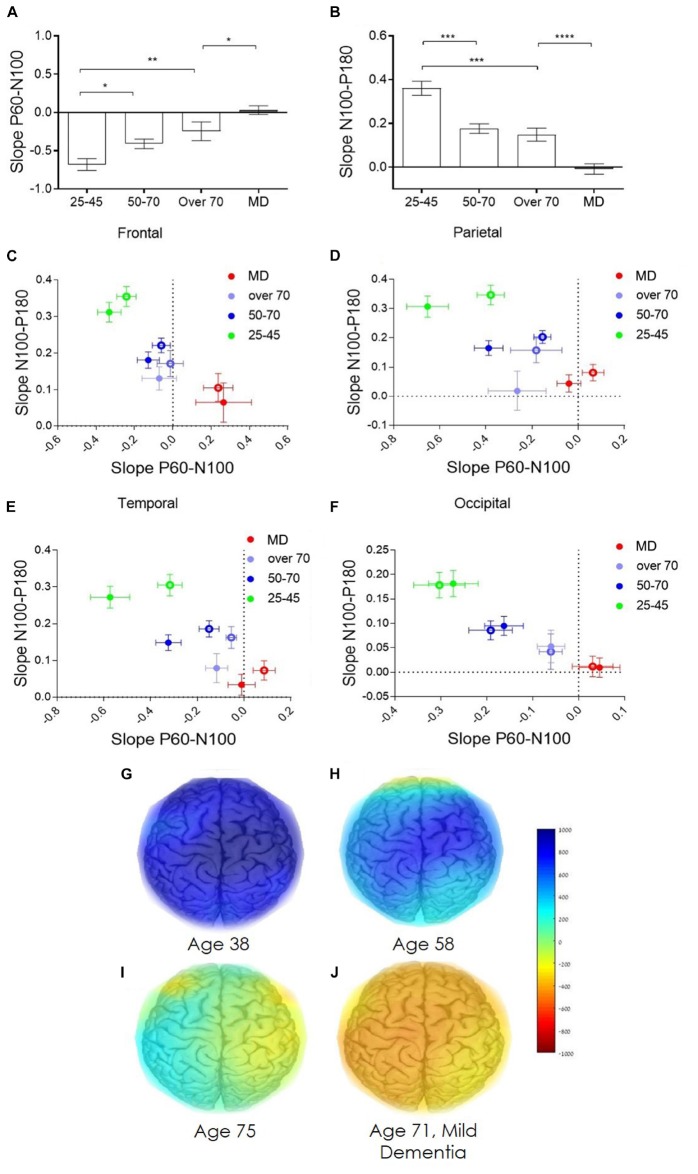
Age dependent network strength. **(A)** Age dependent change in the early slope of evoked response (P60-N100). **(B)** Age dependent change in the late slope of evoked response (N100-P180). **(C–F)** Group average of the ratio between the early and late slopes of response measured from the frontal, parietal, temporal, and occipital cortex electrodes, respectively. **(C–F)** green dots represent the averaged young, 25–45 years old, healthy individuals. Blue dots represents the averaged healthy, 50–70 years old subjects. Purple dots represent the averaged healthy subjects, over 70 years old. Red dots represent patients diagnosed with mild dementia, over 70 years old. Full dots, left hemisphere; empty circles, right hemisphere. **(G–J)** Represent the color-coded images of representative subjects from the four study groups **(G)** 38 years old healthy subject. **(H)** 58 years old healthy subject. **(I)** 75 years old healthy subject. **(J)** 71 years old subject diagnosed with mild dementia. Color coded scale bar represent the ratio between the late slope and the early slope of evoked response.

Direct electrophysiological imaging pinpoints two features that display a significant age dependent decrease in both early (Figure 4A) and late (Figure 4B) components of evoked response (data displayed for right hemisphere, contralateral to stimulation, parietal cortex). Moreover, comparing the two age comparable groups of healthy elderly and patients diagnosed with MD, reveals a significant difference in both components, particularly in the late slope of evoked response (Figure 4B). Group averaged regional ratio between these two slopes (early and late) of evoked response displays a significant, age dependent, change with pronounced differentiation between normal healthy aging, and MD, over the frontal, parietal, temporal and occipital cortical areas (Figures 4C–F, respectively). These extracted cortical network ratio values may be displayed as individual pseudo-color-coded images. Figures 4G–J presents color-coded images of subjects from the representative four study groups. 38-year-old healthy subject demonstrates high and uniform ratio between the late and early slope of evoked response reflected as a homogeneous blue colored brain (Figure 4G). A decline in the measured ratio is demonstrated with age, translated into light blue colored brain of a 58-year-old subject, representing the healthy 50–70 age group (Figure 4H), and a green-yellow colored brain presenting the over 70 years old group (Figure 4I). The MD group, represented by a 71 years-old subject, display a negative high ratio between late and early slopes of evoked response, reflected as orange colored cortical brain network functionality (Figure 4J).

### Age Dependent Changes in Network Short Term Plasticity

**FIGURE 5:**
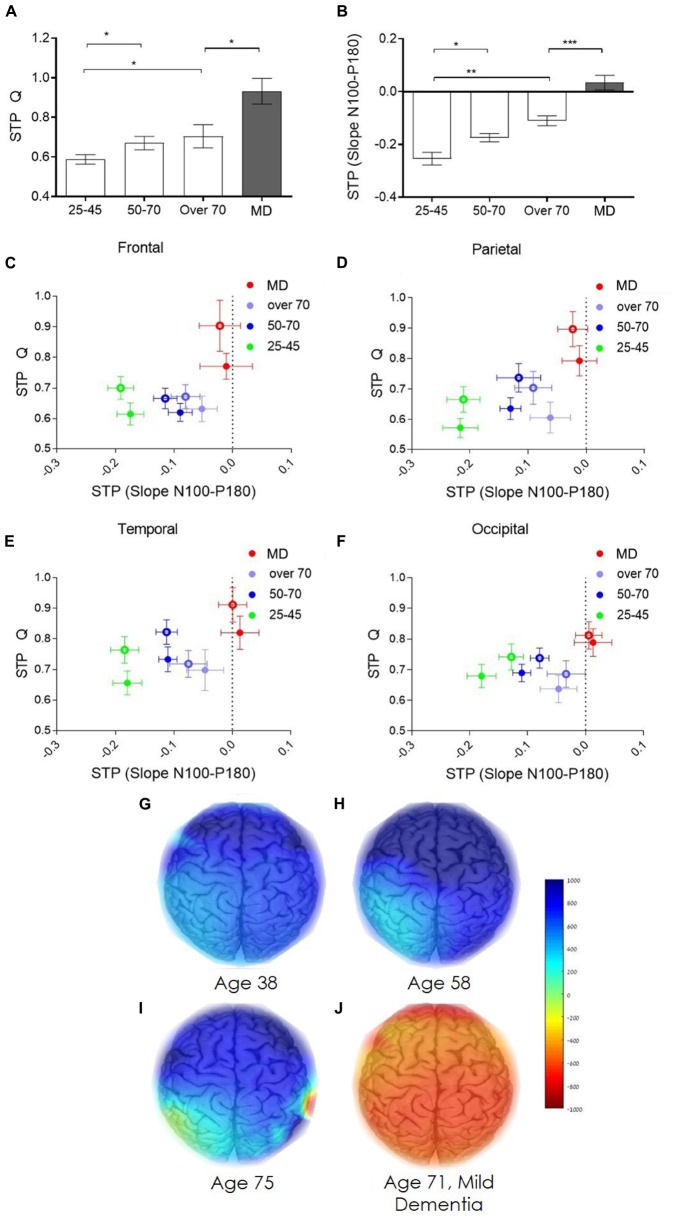
Age dependent network short term plasticity. **(A)** Age dependent short-term plasticity of evoked response charge transfer (STP-Q). **(B)** Age dependent short-term plasticity of evoked response late slope component (STP-slope N100-P180). **(C–F)** Group average of the ratio between STP of the total charge transfer and STP of the late slope of evoked response measured from the frontal, parietal, temporal, and occipital cortex electrodes, respectively. **(C–F)** green dots represent the averaged young, 25–45 years old, healthy individuals. Blue dots represents the averaged healthy, 50–70 years old subjects. Purple dots represent the averaged healthy subjects, over 70 years old. Red dots represent subject diagnosed with mild dementia, over 70 years old. Full dots, left hemisphere; empty circles, right hemisphere. **(G,H)** represent the color-coded images of representative subjects from the four study groups (g) 38 years old healthy subject. **(H)** 58 years old healthy subject. **(I)** 75 years old healthy subject. **(J)** 71 years old subject diagnosed with mild dementia. Color coded scale bar represent the ratio between the STP-Q and STP-slope N100-P180.

Brain network plasticity is known to change with age (Cabeza et al., 2002; Buckner et al., 2008; Wang et al., 2010). Moreover, progression of degenerative disorders as AD, correlate with decrease in brain network plasticity (Palop et al., 2007; Kumar et al., 2017). As TMS-EEG technology has been extensively shown to enable measuring of excitability and plasticity changes in healthy and pathological condition (Tremblay et al., 2019) it provides a platform for such evaluation DELPHI Analysis of the history dependency identified two physiological parameters of network functionality that best distinguished between groups (healthy aging and MD). The ratio between the total charge transfer of response (Q) evoked to an inhibitory protocol of stimulation (STP-Q), and the ratio between the late slope component of evoked response to an inhibitory protocol of stimulation (STP-slope N100-P180) (Figures 5A,B). A significant age dependent increase in the charge transfer STP and a decrease in the late slope STP is observed (Figures 5A,B data from the right hemisphere (contralateral to stimulation) parietal cortex is displayed). Comparing the two age comparable groups of healthy elderly and patients with MD, reveals a significant difference in both parameters. Interestingly, the most significant change in STP between the two groups is observed in the STP of the late slope, in which the MD group displays positive values (Figure 5B) reflecting a significant change in inhibitory response (*P* < 0.001). Group averaged regional ratio between these analyzed parameters of evoked response (Figures 5C–F), displays a pronounced differentiation between normal, healthy aging, and early phases of dementia, over the frontal, parietal, temporal and occipital cortical areas (Figures 5C–F, respectively). Intriguingly, the ratio between these STP calculated parameters demonstrates lower age dependency. These extracted cortical plasticity ratio values may be displayed as individual pseudo-color-coded images. Figures 5G–J, displays the significant differentiation between normal, healthy aging and early stages of dementia, implicating that while single pulse analysis of evoked response demonstrates strong correlation with normal aging (Figure 4), plasticity measures seem to provide a robust parameter for separating normal from abnormal- pathological aging, as in the current case of early stages of dementia (Figure 5).

## Discussion

Current study results display the ability of DELPHI using TMS-EEG technology for measuring crucial brain network parameters of connectivity and plasticity and its relevance for monitoring brain health. Network connectivity measures displayed in this study, indicate monitorable changes that occur with age and point to the ability of this technology to monitor subtle structural and functional changes, as well as the ability to differentiate normal and abnormal aging. Connectivity maps display changes in connectivity between healthy and MD subjects mainly relating frontal areas, indicating a decrease in inter-hemispheric synchronicity, as well as decreased synchronicity between frontal and temporal or parietal areas (Figure 3).

These results are consistent with several structural and functional studies demonstrating intercortical disconnect such as changes in the corpus callosum (CC) in early stages of AD and MCI (Di Paola et al., 2010a, b; Frederiksen et al., 2011). Changes in transcallosal connectivity have also been displayed using TMS in a study differentiating between demented and cognitively impaired non-demented patients (Lanza et al., 2013). TEP slopes, which provide a description of TEP form and an excitation/inhibition reference (Rossi et al., 2009; Tremblay et al., 2019), display an age dependent decrease in both early and late slopes of response (Figure 4). This decrease may be associated with atrophy of gray and white matter or changes in excitation/inhibition balance as supported by anatomical MRI and EEG studies which indicate reduced fiber tracks in frontal and temporal areas and front-occipital reduced synchronicity (Sexton et al., 2011; Dipasquale and Cercignani, 2016; Teipel et al., 2016). In addition, TEP slopes display a clear separation of pathological MD group from healthy control which includes a phase shift represented by slope changes, these may be accounted by severe brain atrophy and/or excitation/inhibition balance shift. Short term plasticity measures (Figure 5), which evaluates the changes in excitation/inhibition balance, are shown to provide discrete parameter which display a sort of binary step function for differentiating the healthy and diseased brain. These results may indicate as to the nature of significant changes in pathological population that results from shifting in excitation/inhibition mechanisms as opposed to connectivity and structural changes that may account for age related changes displayed here. This study results support the significance and value of TMS in understanding and monitoring brain health and pathological aging including neurodegenerative disorders such as Alzheimer’s disease (AD) and vascular dementia. Studies of connectivity, excitability and plasticity utilizing TMS have provided evidence suggesting cortical excitability changes in the early stages of the disease, as well as altered cortical inhibition and cholinergic mechanisms (Bella et al., 2013, 2016; Ni and Chen, 2015; Ferreri et al., 2017b; Lanza et al., 2017). It has also been shown that TMS-EEG evoked potentials (TEP) poses major advantages as: (A) High reproducibility of evoked response within individuals over occipital, parietal, premotor, motor and prefrontal regions (Lioumis et al., 2009; Casarotto et al., 2010; Kerwin et al., 2018). (B) Ability to measure TEP at sub MT intensities. Stimulating the M1 at intensities as low as 40% of the MEP threshold, exemplifying the sensitivity of the measure (Komssi et al., 2004; Komssi and Kähkönen, 2006). (C) Recorded both locally, and in distal electrodes, allowing for the study of the spreading of activation over cortical areas (Ilmoniemi et al., 1997; Komssi et al., 2002).

In this study, we introduce a new scientific and methodological approach, which can be used in the clinical environment, enabling healthcare providers with a bedside tool for the evaluation and monitoring of brain functional status in health and disease. Study results indicate the ability of DELPHI to clinically monitor brain structural and functional changes that may be associated with multiple pathologies, however, this study did not consider different dementia sub groups of AD and Vascular dementia or its precursor of MCI (mild cognitive impairment) and SVD (small vessel disease) this should be further explored in larger pathological populations including different dementia types and their precursor conditions, alongside longitudinal aging studies that might indicate early detection, and exploration of other pathologies.

## Conclusion

The extent of functional changes during brain aging varies among individuals in a way that cannot be quantified using current available clinical tools. Early identification of abnormal brain aging is extensively researched, scanning genetic, biochemical, and neuropsychological aspects of the transition from normal to pathologic aging. Our findings support the notion that evaluating elecro-physiological properties of connectivity and plasticity enable the characterization of age dependent brain functional changes and the monitoring of abnormal aging processes as presented in previous studies. Data presented in this work DELPHI as clinically effective in evaluating brain functionality and may ultimately provide a clinical tool for monitoring brain network function and brain health. DELPHI automated acquisition and analysis system can be used in order to monitor brain health throughout aging and may enable early detection of abnormal pathophysiological changes leading to neurodegeneration, as for the case of MD.

## Data Availability

All datasets generated for this study are included in the manuscript and/or the Supplementary Files.

## Ethics Statement

This study was carried out in accordance with the recommendation of “Assaf-Harofeh” Medical Center Review Board. Protocol was approved by the local institutional “Ethical Committee.” All subjects gave written informed consent in accordance with the Declaration of Helsinki.

## Author Contributions

NZ determined the study design and criteria with OL-L, HF, and ID, and conducted most of the laboratory works. OL-L and HF designed most of the DELPHI software algorithm. HF and ID contributed in designing and writing most of the manuscript and submitting the manuscript for publication. SE was principal investigator in this study. GS was responsible for the cognitive evaluation conducted in the study. DT, SE, and GS contributed in assuring the methods and the quality of the results with reviewing the manuscript, and approving for publication of the content.

## Conflict of Interest Statement

NZ, OL-L, DT, HF, and ID have financial conflicts of interest with QuantalX Neuroscience.

The remaining authors declare that the research was conducted in the absence of any commercial or financial relationships that could be construed as a potential conflict of interest.
