# Supplementary material for: Introducing a Novel Approach for Evaluation and Monitoring of Brain Health Across Life Span Using Direct Non-invasive Brain Network Electrophysiology
Source: Front Aging Neurosci. 2019 Sep 9;11:248. doi: 10.3389/fnagi.2019.00248 (PMC6745309; doi:10.3389/fnagi.2019.00248)
Supplement: Supplementary file 1 [file Data_Sheet_1.docx]

Supplementary Material

DELPHI measurement reliability: Sham stimulation did not result in the appearance of the expected positive and negative peaks on response and displayed no frequency dependent behavior, indicating reliability of measured parameters and therefore mechanisms of network short term plasticity (Supplementary fig.1A-E) (N=5).


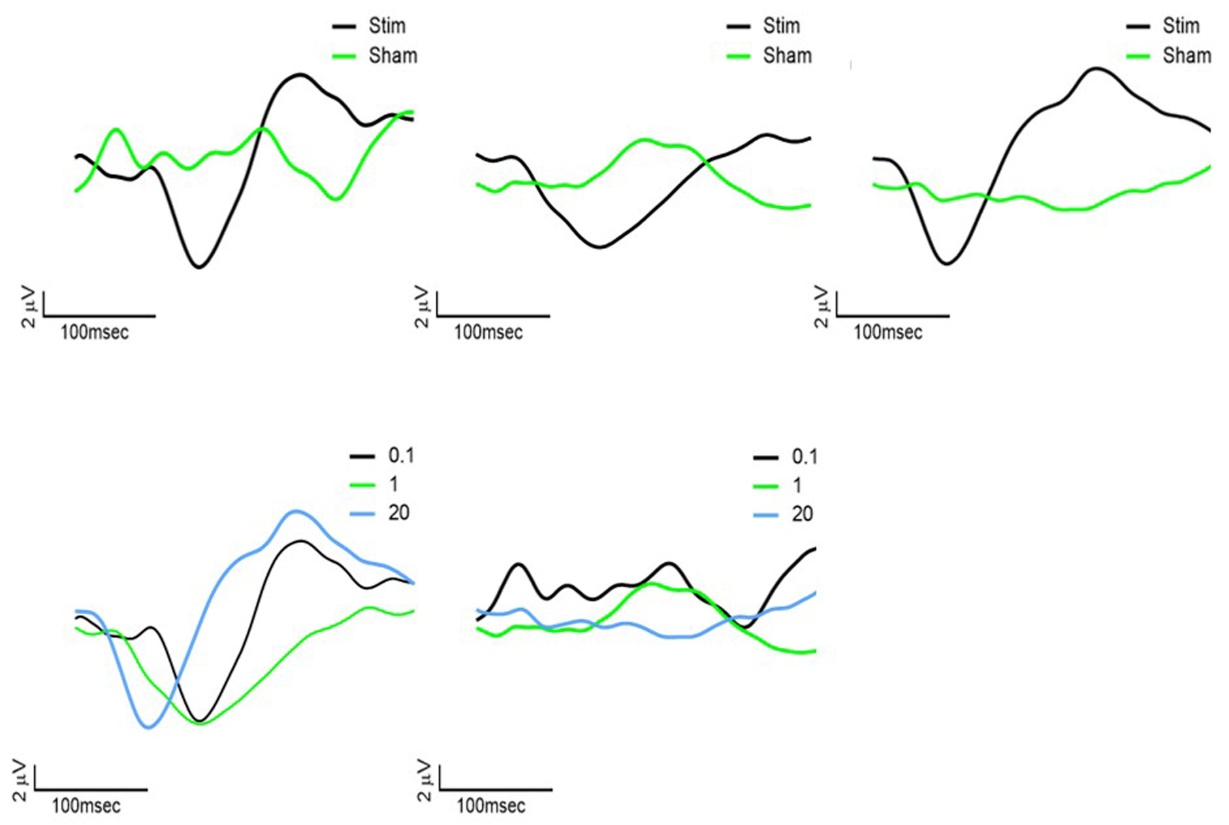


C.

B.

A.

E.

D.

**Supp.fig.1: Sham Controlled TMS evoked stimulation**. A. Single pulse (0.1Hz) representative average traces of left M1 hotspot in healthy subjects. *Black- real stimulation; Green -sham stimulation; B. Inhibitory stimulation (1Hz) representative grand average traces of left M1 hotspot in healthy subjects. *Black- stimulation; Green -Sham; C. Excitatory (20Hz) representative grand average traces of left hotspot healthy subjects. *Black- stimulation; Green -Sham; D. representative grand average traces of real TMS stimulation at 0.1Hz, 1Hz, 20 Hz (black, green, blue respectively) over the left M1 hotspot. E. representative grand average traces of sham TMS stimulation at 0.1Hz, 1Hz, 20 Hz (black, green, blue respectively) over the left M1 hotspot. (N=5, P<0.01).

Electrophysiological features of TMS evoked EEG response were defined. A single pulse TMS results in a sequence of positive and negative amplitudes at specific timing following stimulation, including positive amplitudes peaks at 60 and 10 msec and negative amplitude peak at 100 msec following stimulation (Supplementary Fig.2A,i,ii, dotted lines mark the specific amplitude peaks timings). Properties of network strength and plasticity were evaluated in the young (25-45 years old) healthy subjects’ group (Supplementary Fig.2A,B) .

**
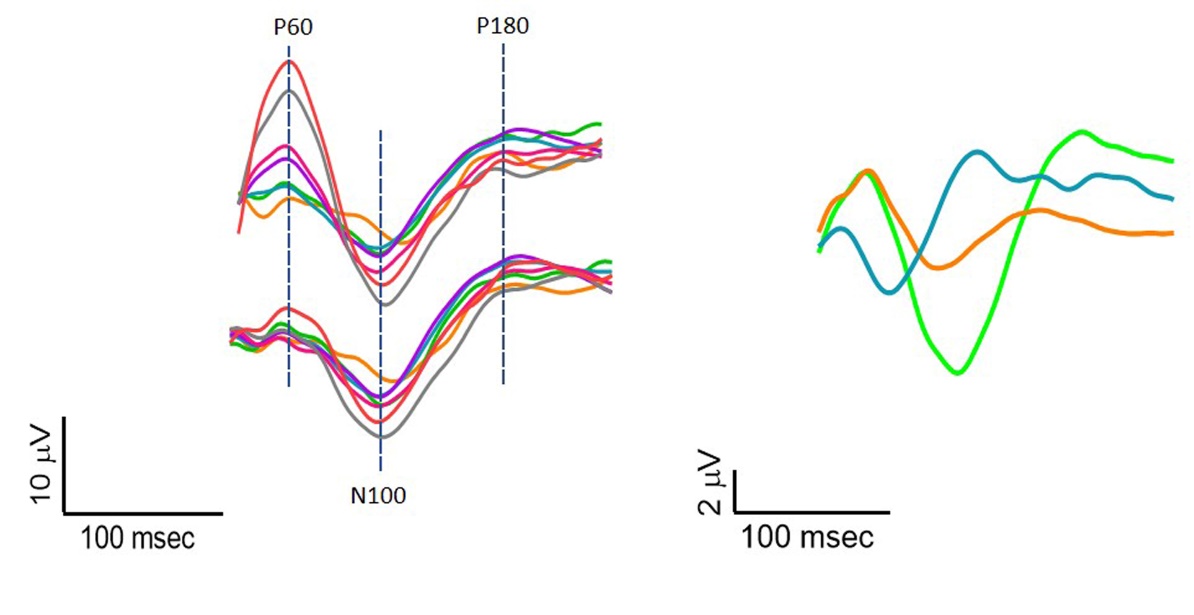
**

B.

A.

i

ii

**Supp.fig.2:** Intensity and frequency dependency of TMS evoked EEG response: (a) Evaluation of network strength (defined as the Input-Output (I/O) ratio of evoked response) demonstrates an increase in the evoked signal response potential at increased stimulation intensities, in both hemispheres (left- ipsilateral to stimulation and right- contralateral to stimulation, Fig.2Ai,ii respectively).dotted lines mark the specific amplitude peaks timings (positive peaks at 60 and 180msec and negative peak 100msec following TMS single stimuli (b). Network short term plasticity was evaluated by changing the frequency of stimulation. Three stimulation frequencies were tested, 0.1Hz (green trace, single pulse, showing no ‘history dependency’ [67], 1Hz red trace, (low frequency– evoking inhibition of response [62, 63] and 20 Hz (blue trace, high frequency– evoking excitation of evoked response [60, 61].


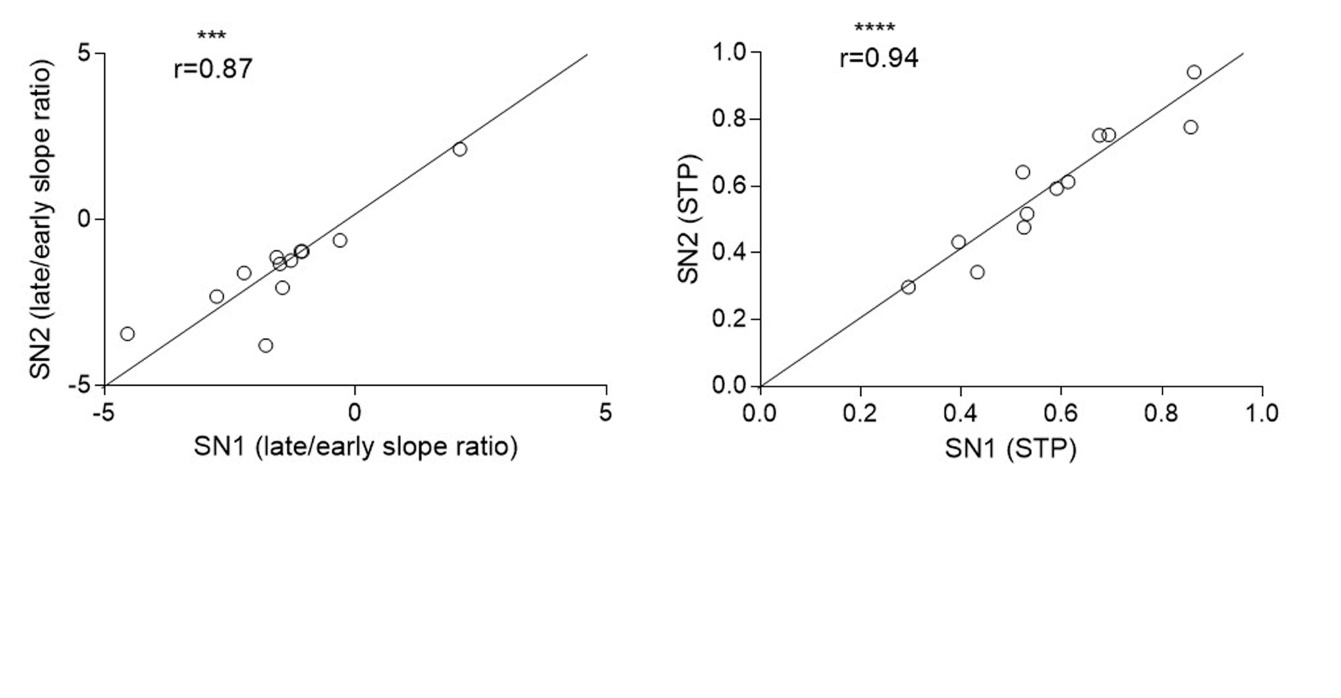
Reproducibility test demonstrate the high reliability and reproducibility of the DELPHI analysis of neuro-physiological parameters displaying reliability coefficient (r) of 0.87 and 0.94.

B.

A.

**Supp.fig.3:** Reliability testing: reliability testing performed on 11 healthy subjects tested 1-2 weeks apart (a) Single pulse response: Correlation of slope ratio parameters between first and second session. Black circles represent healthy subjects. Pearson’s r=87, p<0.001; (b). History dependent plasticity: Correlation of STP ratio parameters between first and second session. Black circles represent healthy subjects. Pearson’s r=9, p<0.0001.
